# Rejoinder: More Limitations of Bayesian Leave-One-Out Cross-Validation

**DOI:** 10.1007/s42113-018-0022-4

**Published:** 2019-01-15

**Authors:** Quentin F. Gronau, Eric-Jan Wagenmakers

**Affiliations:** University of Amsterdam, Nieuwe Achtergracht 129 B, 1018 WT Amsterdam, The Netherlands

**Keywords:** Bayesian stacking, Bayes factor, Bayesian model averaging, Prequential approach, M-open

## Abstract

We recently discussed several limitations of Bayesian leave-one-out cross-validation (LOO) for model selection. Our contribution attracted three thought-provoking commentaries. In this rejoinder, we address each of the commentaries and identify several additional limitations of LOO-based methods such as Bayesian stacking. We focus on differences between LOO-based methods versus approaches that consistently use Bayes’ rule for both parameter estimation and model comparison. We conclude that LOO-based methods do not align satisfactorily with the epistemic goal of mathematical psychology.

Bayesian leave-one-out cross-validation (LOO) is increasingly popular for the comparison and selection of quantitative models of cognition and behavior.^1^ In a recent article for *Computational Brain & Behavior*, we outlined several limitations of LOO (Gronau and Wagenmakers this issue). Specifically, three concrete, simple examples illustrated that when a data set of infinite size is perfectly in line with the predictions of a simple model $\mathcal {M}_{S}$ and LOO is used to compare $\mathcal {M}_{S}$ to a more complex model $\mathcal {M}_{C}$, LOO shows bounded support for $\mathcal {M}_{S}$. As we mentioned, this model selection inconsistency has been known for a long time (e.g., Shao 1993). We also discussed limitations that were unexpected (at least to us). Concretely, for data perfectly consistent with the simpler model $\mathcal {M}_{S}$, (1) the limiting bound of evidence for $\mathcal {M}_{S}$ is often surprisingly modest; (2) the LOO preference for $\mathcal {M}_{S}$ may be a nonmonotonic function of the number of observations (meaning that additional observations perfectly consistent with $\mathcal {M}_{S}$ may in fact *decrease* the LOO preference for $\mathcal {M}_{S}$); and (3) contrary to popular belief, the LOO result can depend strongly on the parameter prior distribution, even asymptotically.

Our discussion of the limitations of LOO attracted three commentaries. In the first commentary, Vehtari et al. (this issue) claim that we “focus on pathologizing a known and essentially unimportant property of the method; and they fail to discuss the most common issues that arise when using LOO for a real statistical analysis.” Furthermore, Vehtari et al.state that we used a version of LOO that is not best practice and they suggest to use LOO-based Bayesian stacking instead (Yao et al. 2018). Vehtari et al. also criticize us for making the assumption that one of the models under consideration is “true” and use this as a springboard to question the usefulness of Bayes factors (e.g., Jeffreys 1961; Kass and Raftery 1995) and Bayesian model averaging (BMA; e.g., Hoeting et al. 1999; Jevons 1874/1913). Finally, Vehtari et al. point out what they believe are more serious limitations of LOO-based methods. The second commentary is by Navarro (this issue) and discusses how the scientific goal of explanation aligns with traditional statistical concerns; Navarro suggests that the model selection literature may focus too heavily on the statistical issues of model choice and too little on the scientific questions of interest. In the third commentary, Shiffrin and Chandramouli (this issue) advocate Bayesian inference for non-overlapping model classes. Furthermore, Shiffrin and Chandramouli advocate tests of interval-null hypotheses instead of point-null hypotheses. Finally, Shiffrin and Chandramouli demonstrate that comparing non-overlapping hypotheses (where the null is an interval) eliminates the model selection inconsistency of LOO.

We thank the contributors for a productive discussion. To keep this rejoinder concise, we decided to address only the key points of disagreement. First, however, we will outline what we believe to be the primary goal of mathematical psychology.

## Mathematical Psychology: An Epistemic Enterprise

Mathematical psychology is founded on the principle that psychological theories about cognition and behavior ought to be made precise by implementing them as quantitative models. Fum et al. (2007, p. 136) write:

“Verbally expressed statements are sometimes flawed by internal inconsistencies, logical contradictions, theoretical weaknesses and gaps. A running computational model, on the other hand, can be considered as a sufficiency proof of the internal coherence and completeness of the ideas it is based upon.”

There exist different opinions about the role of models. As mentioned by Navarro (this issue), Bernardo and Smith (1994, p. 238) state:

“Many authors [...] highlight a distinction between what one might call *scientific* and *technological* approaches to models. The essence of the dichotomy is that scientists are assumed to seek *explanatory* models, which aim at providing insight into and understanding of the “true” mechanisms of the phenomenon under study; whereas technologists are content with *empirical* models, which are not concerned with the “truth” but simply with providing a reliable basis for practical action in predicting and controlling phenomena of interest.”

Bernardo and Smith (1994, p. 238) conclude that when models are evaluated based on their predictions, the distinction is immaterial. In contrast, we believe that the distinction remains crucial. To us, the purpose of mathematical psychology is epistemic: the ultimate goal is to understand phenomena by developing theories, implementing these theories rigorously as quantitative models, and testing these models on observed data. Hence, our view of mathematical psychology aligns with what Bernardo and Smith call the “scientific approach.” In contrast, the main goal of the “technological approach” is the prediction of future data. There is an important distinction between these two approaches since, if the goal is solely prediction, one may be satisfied with models and methods that can be characterized as black-box “prediction devices.” The components and parameters of such prediction devices may not permit a substantive interpretation.

We believe that for many mathematical psychologists predictive adequacy is only a pragmatic means to an epistemic end. Quantitative models of cognition and behavior typically feature parameters that represent latent cognitive processes; these are of interest in and of themselves and do not serve only as tuning knobs of prediction devices. We do not wish to suggest that prediction is unimportant; in fact, we believe that models ought to be compared based on the predictions they made for observed data. However, we feel that the goal in mathematical psychology is virtually always an epistemic one, where models instantiate meaningful theories, and not a predictive one, where predictions are made for their own sake without the goal of developing and employing substantive theory. The following sections demonstrate by example that LOO-based methods have important limitations when the goal is epistemic rather than purely predictive.

## Rejoinder to Vehtari, Simpson, Yao, & Gelman

Vehtari et al. (this issue, henceforth, VSYG) claim that we used a LOO version that is not in line with best practice and conclude that “[..] the claimed “limitations of Bayesian leave-one-out cross-validation” from GW do *not* apply to the version of Bayesian leave-one-out cross-validation that we recommend.” Specifically, (1) VSYG claim that we fail to take into account the empirical variance of the LOO estimate; they recommend doing so by using pseudo-BMA + weights (Yao et al. 2018); (2) VSYG suggest that it would be even better to use Bayesian stacking (Yao et al. 2018). First, we agree that one should take into account the empirical variance of the LOO estimate in case it is nonzero. However, as VSYG mention “[...] this does not make a difference in their very specialized examples.” Second, since VSYG claim that the limitations, we mentioned are well-known and suggest Bayesian stacking instead, below we outline further limitations of LOO-based methods such as Bayesian stacking. We start by discussing the relevance of the assumption that one of the models under consideration is “true” which VSYG use to question the usefulness of Bayes factors and Bayesian model averaging.

### LOO is Motivated by an Illusory Distinction Between $\mathcal {M}$-Open Tools and $\mathcal {M}$-Closed Tools

LOO-based methods have been recommended for what is called the $\mathcal {M}$-open setting (Bernardo and Smith 1994). Consider a set of *M* candidate models: $\mathcal {M}_{1}, \mathcal {M}_{2}, \ldots , \mathcal {M}_{M}$. $\mathcal {M}$-open refers to a situation where the “true” model is not included in the set of candidate models. This stands in contrast to the $\mathcal {M}$-closed setting where one of the models in the set is “true” in the sense that it corresponds to the data-generating process.

In the $\mathcal {M}$-closed case it is valid (although not universally recommended; see Gelman et al. 2014, chapter 7.4; Gelman and Shalizi 2013) to employ model comparison and prediction approaches that consistently use Bayes’ rule, not only to update one’s knowledge about parameters within a model, but also about the models themselves (e.g., by means of BMA, Bayes factors, posterior model probabilities). These approaches assign prior probabilities $p(\mathcal {M}_{k})$, *k* = 1,2,…,*M* to a set of *M* models.^2^

In the $\mathcal {M}$-open case, however, the appropriateness of these supposedly “$\mathcal {M}$-closed tools” is often questioned (Bernardo and Smith 1994, pp. 383–407; Yao et al. 2018). Moreover, George Box’s famous adage “all models are wrong” may then be invoked to question the use of these “$\mathcal {M}$-closed tools” in any practical application. For instance, Li and Dunson (2016) argue that “Philosophically, in order to interpret $\text {pr}(\mathcal {M}_{j} \mid y^{(n)})$ as a model *probability*, one must rely on the (arguably always flawed) assumption that one of the models in the list $\mathcal {M}$ is exactly true, known as the $\mathcal {M}$-closed case.”

Our objections to this line of reasoning are threefold. First, if we were to accept that these “$\mathcal {M}$-closed tools” are unsuitable for practical data analysis, this would similarly disqualify the specification of parameter priors and the computation of posterior predictives. As explained in the next section, individual parameter values or specific parameter ranges can be conceptualized as individual models.

Second, Bayes’ rule does not refer to an underlying ”truth” and the prior probability that is assigned across models (or across parameters) quantifies *relative* plausibility. Feldman (2015) has emphatically argued this point:^3^

“But such a strong assumption [that one of the candidate models is true] is not really necessary in a Bayesian framework—at least, it is not required or implied by any of the equations. Rather, Bayesian inference only assumes that there is some set *M* of possible models under consideration, which are tied to the data via likelihood functions *p*(*X*|*M*). Bayes’ rule allows these models to be compared *to each other* in terms of plausibility, but says nothing whatsoever about whether any of the models is true in a larger or absolute sense (see Feldman, 2014). The ‘truth’ of the models (whatever that even means–see remarks above about semantics) never enters into it.” (Feldman 2015, p. 1524)

Third, Feldman (2013, pp. 17–18) points out, as did Bayesian pioneers Ed Jaynes and Dennis Lindley before him, that the assignment of prior probabilities is always conditional on background knowledge $\mathcal {K}$. Hence, when we write $p(\mathcal {M}_{k})$ this is really just a convenient shorthand for the more accurate notation $p(\mathcal {M}_{k} \mid \mathcal {K})$, a renormalized probability for a subset of relevant models selected by conditioning on the current knowledge $\mathcal {K}$. Background knowledge $\mathcal {K}$ provides the pragmatic filter that allows us to define, from the infinite collection of possible models, a subset of models that pass a certain minimum threshold of plausibility, feasibility, or substantive interest. This conceptualization of prior model probabilities is in line with our epistemic view on mathematical psychology. Given a set of competing theoretical accounts of interest, implemented as quantitative models (i.e., given our background knowledge $\mathcal {K}$), we are interested in quantifying the relative evidence for each of these models based on observed data. Nowhere do we assume any of the models that represent rival theories to be true in an absolute sense.

We do not wish to suggest that the possibility of model-misspecification can be happily ignored; all models necessarily make assumptions and simplifications and it may happen that given a set of models, even the best one fails to provide a satisfactory description of the phenomenon of interest. In our opinion, however, this does *not* suggest that the entire approach of assigning prior probabilities to a set of rival models is flawed from the outset or that it does not make sense. In contrast, the presence of model-misspecification suggests that one ought to refine the models or develop new theories that are able to better capture the relevant aspects of the phenomenon of interest (i.e., expand the background knowledge base $\mathcal {K}$). These new model versions can then be incorporated in the set of models and can be compared to each other based on new data.

### LOO Depends on an Arbitrary Distinction Between Parameter Estimation and Model Comparison

We do not believe that the distinction between $\mathcal {M}$-open and $\mathcal {M}$-closed is a valid argument against approaches that consistently use Bayes’ rule for both parameters and models. Those who disagree may feel that assigning model probabilities $p(\mathcal {M}_{k})$ does not make sense in the $\mathcal {M}$-open setting; these dissenters would, in our opinion, then also need to object to assigning prior probabilities to parameters and computing quantities such as posterior predictives. The reason is that the distinction between parameter estimation and model comparison can be regarded as artificial (see also Gelman 2011, p. 76). It has long been known that estimation can be viewed as a special case of model comparison (also known as ‘testing’):^4^

“We shall not consider the philosophy of Bayesian estimation procedures here. These procedures can be regarded as a special case of Bayesian hypothesis testing since every statement of the form that a vectorial parameter belongs to a region is itself a hypothesis [but estimates are less often formulated before making observations].” (Good 1983, p. 126)

#### Discrete Parameters

**Fig. 1:**
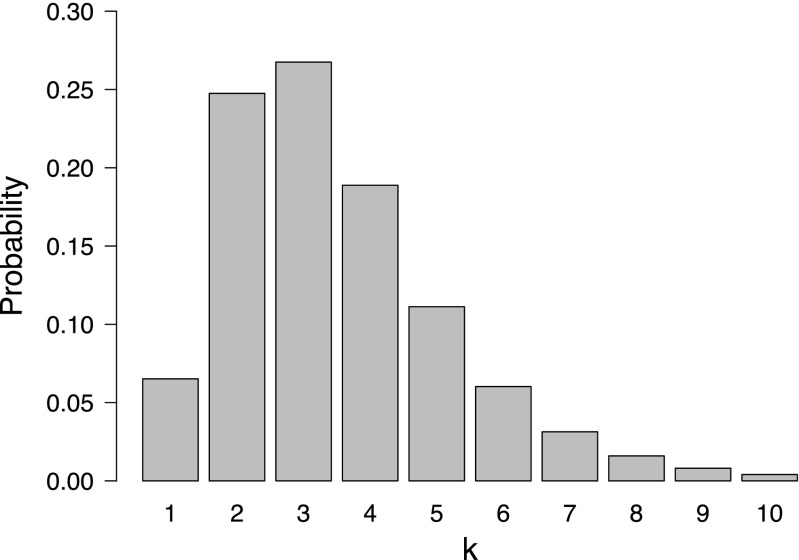
Parameter estimation or model comparison? Shown is the posterior distribution for the tumor transplant example based on 1 “take” out of 6 attempts and a uniform prior for *k*, the number of genes determining transplantability. Here, *k* may be regarded as a parameter, such that the depicted distribution is a parameter posterior distribution, or *k* may be regarded as indexing separate models, so that the depicted distribution corresponds to posterior model probabilities. Available at https://tinyurl.com/y94uj4h8 under CC license https://creativecommons.org/licenses/by/2.0/

The fact that labeling a problem as parameter estimation or model comparison can be regarded as arbitrary is most apparent for discrete parameter models. As a concrete example, consider a scenario inspired by Hammersley (1950, p. 236) about tumor transplantability in mice (see also Choirat and Seri 2012). For a certain type of mating, the probability of a tumor “taking” when transplanted from one of the grandparents is (1/2)^*k*^, where *k* is an integer that corresponds to the number of genes determining transplantability (all of which must be present for a “take” to occur). Suppose, for illustrative purposes, we know that the number of relevant genes is between 1 and 10 and we deem each number equally likely a priori: *p*(*k*) = 1/10, for all *k* ∈{1,2,…,10}. The likelihood corresponds to a binomial distribution with success probability *𝜃* = (1/2)^*k*^. Suppose fictitious data show 1 “take” out of 6 attempts. The resulting posterior distribution for *k* is displayed in Fig. 1.

In this example, *k* could be regarded as a parameter, so that the distribution in Fig. 1 is a parameter posterior distribution. However, *k* could also be regarded as an index for a set of 10 competing models $\mathcal {M}_{1}, \mathcal {M}_{2}, \ldots , \mathcal {M}_{10}$, where $\mathcal {M}_{k}: \theta = (1/2)^{k}$, *k* = 1,2,…,10. In this case, the distribution in Fig. 1 visualizes the posterior model probabilities.

After having obtained a posterior over the number of genes *k*, one may be interested in predicting new data *y*_new_ given the observed data *y* (i.e., 1 “take” out of 6 attempts). This is achieved by marginalizing over *k*:

1 $$ p(y_{\text{new}} \mid y) = \sum\limits_{k = 1}^{10} p(y_{\text{new}} \mid k) p(k \mid y),  $$

where *p*(*k*∣*y*) corresponds to the posterior distribution depicted in Fig. 1. When *k* is regarded as a parameter, Eq. 1 corresponds to the posterior predictive distribution; when *k* is regarded as indexing separate models, Eq. 1 corresponds to the BMA predictive distribution for new data. This shows that the mathematical operation of computing a posterior predictive is identical to that used in Bayesian model averaging.^5^ Proponents of LOO-based methods who believe there is an issue with BMA may not appreciate that this issue applies with equal force to posterior predictives, a concept integral to LOO-based methods such as Bayesian stacking. When treating *k* as a parameter, one could equally ask “what if none of the values for *k* is ‘true’? How can we define *p*(*k*) in the knowledge that none of these values will perfectly capture the data-generating process?”

As mentioned earlier, one may argue that it *does* make sense to define *p*(*k*), even when it is not strictly speaking true, because we assume that we operate within a more narrow context, one that is obtained by conditioning on a model $\mathcal {M}_{\text {Estimation}}$:^6^ $p(k \mid \mathcal {M}_{\text {Estimation}})$. We agree and, crucially, this conditioning argument applies to models as well; we should really write $p(\mathcal {M}_{k} \mid \mathcal {K})$, that is, the probability of model $\mathcal {M}_{k}$ given background knowledge $\mathcal {K}$. Both for parameters and models, plausibility assessments are always part of a subset of possibilities. In other words, regardless of whether we are estimating parameters or comparing models, we have to make assumptions and simplifications. When these assumptions are violated this signals a potential problem with the inference, but it does not mean that the entire approach is flawed from the outset. In sum, for predictions from discrete parameter models the proponents of LOO may recommend posterior predictives when the problem is phrased as estimation, whereas they may recommend LOO-based Bayesian stacking when the problem is phrased as model comparison.

#### Continuous Parameters

We have argued that the distinction between parameter estimation and model comparison is purely semantic. Bayes’ rule does not care about such labels: the same result is obtained regardless of what is called a parameter or a model. In contrast, LOO-based methods lack this coherence: the distinction between parameters and models is crucial. For instance, BMA yields the same results as Bayesian parameter estimation when the set of models is obtained by partitioning a continuous parameter space into non-overlapping intervals, with prior model probabilities set equal to the prior mass in the respective intervals (see Appendix B for a derivation). As a concrete example, suppose observations *y*_*i*_, *i* = 1,2,…,*n* are assumed to follow a Bernoulli distribution with success probability *𝜃*. In this scenario, one could assign *𝜃* a prior distribution *p*(*𝜃*)—for concreteness, we assume a uniform prior—and then obtain a posterior for *𝜃*. Subsequently, one may obtain predictions for a new data point *y*_new_ based on the posterior for *𝜃*. Alternatively, one could also use BMA for the following three models: $\mathcal {M}_{1}: \theta \in [0, .25)$, $\mathcal {M}_{2}: \theta \in [.25, .75]$, and $\mathcal {M}_{3}: \theta \in (.75, 1]$. Given a uniform prior on *𝜃*, BMA and Bayesian parameter estimation yield identical results when (1) the prior for *𝜃* under each model is a (renormalized) uniform prior and (2) the prior model probabilities are $p(\mathcal {M}_{1}) = .25$, $p(\mathcal {M}_{2}) = .5$, and $p(\mathcal {M}_{3}) = .25$ (i.e., the probabilities that the uniform prior for *𝜃* assigns to the three intervals).

**Fig. 2:**
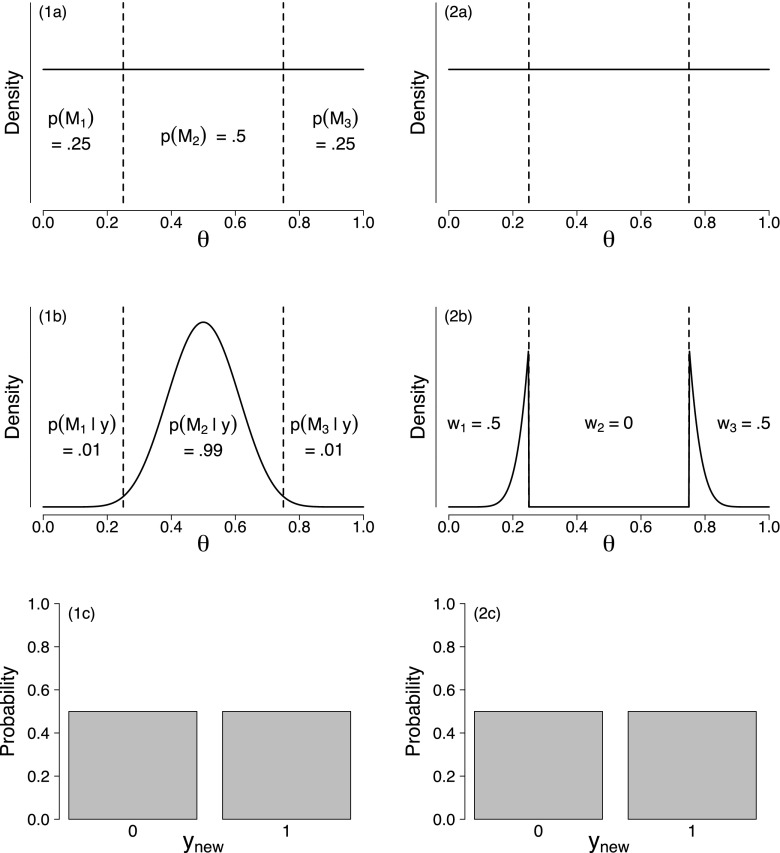
BMA (left column) and Bayesian stacking (right column) results for the Bernoulli example based on 10 successes out of *n* = 20 observations. Panels (1a) and (2a) show the uniform prior distribution for *𝜃* which is partitioned into three non-overlapping intervals to yield models $\mathcal {M}_{1}$, $\mathcal {M}_{2}$, and $\mathcal {M}_{3}$. Panel (1a) also displays the prior model probabilities (not used in stacking). Panel (1b) displays the BMA posterior based on using the posterior model probabilities as averaging weights, and panel (2b) displays a model-averaged posterior obtained using the stacking weights. Panel (1c) displays the BMA predictions for a single new observation *y*_new_ and panel (2c) displays the corresponding predictions from stacking. Available at https://tinyurl.com/yaql2vt4 under CC license https://creativecommons.org/licenses/by/2.0/

The left column of Fig. 2 displays the BMA results for *n* = 20 observations, half of which are successes. Panel (1a) depicts the uniform prior distribution for *𝜃* that is partitioned into three intervals to produce the models $\mathcal {M}_{1}$, $\mathcal {M}_{2}$, and $\mathcal {M}_{3}$. The displayed prior model probabilities correspond to the mass that the uniform prior for *𝜃* assigns to each interval. Panel (1b) displays the BMA posterior distribution—it is identical to the posterior obtained when conducting Bayesian parameter estimation for the common model that assigns *𝜃* a uniform prior from 0 to 1. The weights that BMA uses to average the results of the different models are given by the posterior model probabilities. $\mathcal {M}_{2}$ receives almost all posterior model probability: $p(\mathcal {M}_{2} \mid y) = .99$, as the observed data are predicted much better by values of *𝜃* that are inside rather than outside the [.25, .75] interval. Panel (1c) displays the BMA predictive distribution for a single new observation *y*_new_. This distribution is identical to the posterior predictive distribution obtained based on Bayesian parameter estimation. In line with the fact that the posterior for *𝜃* is symmetric around .5, *y*_new_ is predicted to be a success with probability .5.

The right column of Fig. 2 displays the results obtained when using Bayesian stacking (Yao et al. 2018). Panel (2a) displays again the uniform prior distribution for *𝜃* that is partitioned into three intervals to produce the models $\mathcal {M}_{1}$, $\mathcal {M}_{2}$, and $\mathcal {M}_{3}$. In contrast to BMA, Bayesian stacking does not assign prior probabilities to the different models. Panel (2b) displays a model-averaged posterior distribution and panel (2c) displays the Bayesian stacking predictive distribution; both of these are obtained by combining the different models according to the stacking weights.^7^ The stacking-based predictions are indistinguishable from those of BMA and appear very reasonable: it is predicted that the next observation will be a success with probability .5. However, the stacking weights themselves are highly undesirable indicators of the plausibility of the different models in light of the observed data. $\mathcal {M}_{2}$, the model that clearly outpredicts the other two, is in fact decisively ruled out, as its stacking weight is equal to 0. To understand this result, first note that the stacking weights *w*_*k*_, *k* = 1,2,…,*M* are obtained by maximizing the following objective function (subject to the constraint that *w*_*k*_ ≥ 0 and ${\sum }_{k = 1}^{M}w_{k} = 1$):

2 $$ \frac{1}{n} \sum\limits_{i = 1}^{n} \log\left( \sum\limits_{k = 1}^{M} w_{k} p(y_{i} \mid y_{-i}, \mathcal{M}_{k})\right). $$

Table 1 displays the LOO predictive density values for *y*_*i*_ = 0 and *y*_*i*_ = 1 for the three models under consideration. $\mathcal {M}_{1}$ and $\mathcal {M}_{3}$ make mirrored predictions, whereas the LOO predictive density for $\mathcal {M}_{2}$ is identical for *y*_*i*_ = 0 and *y*_*i*_ = 1. Combining the models’ LOO predictive densities according to the stacking weights *w*_1_ = .5, *w*_2_ = 0, and *w*_3_ = .5 yields ${\sum }_{k = 1}^{M} w_{k} p(y_{i} \mid y_{-i}, \mathcal {M}_{k}) \approx .4982$, for all *i* = 1,2,…,*n*. The objective function thus attains a larger value than when using, for instance, *w*_1_ = 0, *w*_2_ = 1, and *w*_3_ = 0 (${\sum }_{k = 1}^{M} w_{k} p(y_{i} \mid y_{-i}, \mathcal {M}_{k}) \approx .4786$), or when using *w*_1_ = 1/3, *w*_2_ = 1/3, and *w*_3_ = 1/3 (${\sum }_{k = 1}^{M} w_{k} p(y_{i} \mid y_{-i}, \mathcal {M}_{k}) \approx .4917$).

**Table 1:** LOO predictive densities

Observation	$p(y_{i} \mid y_{-i}, \mathcal {M}_{1})$	$p(y_{i} \mid y_{-i}, \mathcal {M}_{2})$	$p(y_{i} \mid y_{-i}, \mathcal {M}_{3})$
*y*_*i*_ = 0	.7758	.4786	.2206
*y*_*i*_ = 1	.2206	.4786	.7758

We need to emphasize that Yao et al. do not suggest to use the stacking weights to obtain a model-averaged posterior as in panel (2b); instead, Yao et al. focus purely on predictions. Nevertheless, this distribution highlights the undesirable nature of the stacking weights when used as indicators for the plausibility of different models and parameters. The plot also shows how Bayesian stacking achieves predictions that are indistinguishable from the BMA predictions by combining two models with low plausibility that make mirrored predictions.

Bayesian stacking was designed to make good predictions in the presence of model-misspecification and may be a valuable tool in case prediction is the main goal. However, we believe that mathematical psychology has an epistemic purpose: researchers are typically interested in quantifying the evidence for different models which represent competing theories of cognition and behavior. Our example illustrates that the stacking weights do not appear to align satisfactorily with this goal. This is also highlighted by the fact that, as VSYG mention, the stacking weight for a simple general law model (i.e., example 1 of Gronau and Wagenmakers this issue) is equal to 1 when all observations are in line with the general law, *independent* of the number of observations *n*. VSYG state: “The lack of dependence on *n* may look suspicious.” Indeed, suppose one is asked whether all swans are white and two white swans are observed. Is it warranted to conclude that the general law is now firmly established? Should predictions about the future disregard the possibility that the general law might fail? Even though VSYG provide an explanation why they believe suspicion is not warranted, we remain doubtful.

In sum, we are skeptical about the usefulness of Bayesian stacking in mathematical psychology where the goal is of an epistemic and not a purely predictive nature.

### LOO Depends on an Arbitrary Distinction Between Data that Arrive Sequentially or “Simultaneously”

LOO is based on repeatedly leaving out one of the observations and evaluating the prediction for this held-out data point based on the remaining observations. Concretely, given data *y* = (*y*_1_, *y*_2_,…,*y*_*n*_), LOO evaluates the predictive density *p*(*y*_*i*_∣*y*_−*i*_) for all *i* = 1,2,…,*n*, where *y*_−*i*_ denotes all data points except the *i* th one. It is well-known that LOO is theoretically unsatisfactory when applied to time series data since, in this case, LOO uses the future to predict the past, for all *i*≠*n* (e.g., Bürkner et al. 2018). As VSYG point out, there exist alternative cross-validation schemes that do not have this property and may be applied in this context (e.g., Bürkner et al. 2018). Therefore, time series data are treated differently from data that do not exhibit a temporal structure. However, we argue that *all* data form a time series. When conducting an experiment, participants come in over time; the data have a temporal order. Consequently, the use of LOO implies that one uses the future to predict the past. It seems unsatisfactory to apply a method that is not recommended for time series to data that have a temporal order, even if that temporal order is disregarded in the analysis because the observations are judged to be exchangeable.

Another consequence of the fact that LOO does not respect the temporal nature of the data is that LOO is inconsistent with what Dawid (1984, p. 278) termed the *prequential approach* which “[...] is founded on the premiss that the purpose of statistical inference is to make sequential probability forecasts for future observations.” In contrast, Bayes factors are consistent with the prequential approach (e.g., Wagenmakers et al. 2006). The reason is that the Bayes factor compares two models based on the ratio of their *marginal likelihoods*. The marginal likelihood corresponds to the joint probability of the data given a model. Consequently, it is easy to show that the marginal likelihood of model $\mathcal {M}_{k}$ can be conceptualized as an accumulation of one-step-ahead predictions:

3 $$\begin{array}{@{}rcl@{}} p(y\!\mid\!\mathcal{M}_{k}) &= &p(y_{1}\!\mid\!\mathcal{M}_{k}) \!p(y_{2} \!\mid\! y_{1}, \mathcal{M}_{k})\\&&p(y_{3} \!\mid\! y_{1:2}, \mathcal{M}_{k}) {\ldots} p(y_{n}\!\mid\! y_{1:(n - 1)}, \mathcal{M}_{k}), \end{array} $$

where *y*_1:*i*_ = (*y*_1_, *y*_2_,…,*y*_*i*_) denotes the first *i* observations. Each term in Eq. 3 is obtained by integrating over the model parameters *𝜃*. For the first observation, $p(y_{1} \mid \mathcal {M}_{k}) = {\int }_{\Theta } p(y_{1} \mid \theta , \mathcal {M}_{k}) p(\theta \mid \mathcal {M}_{k}) \text {d} \theta $, and for *i* > 1, $p(y_{i} \mid y_{1:(i - 1)}, \mathcal {M}_{k}) = {\int }_{\Theta } p(y_{i} \mid \theta , y_{1:(i-1)}, \mathcal {M}_{k}) p(\theta \mid y_{1:(i-1)}, \mathcal {M}_{k}) \text {d} \theta $. Thus, Bayes factors—but not LOO—produce the same result, regardless of whether the data are analyzed one at a time or all at once.

A common criticism of the Bayes factor is its dependence on the parameter prior distribution since one starts by making predictions based on the prior distribution. There are a number of replies to this concern. First, it may be regarded as desirable that the result depends on the prior information, as this allows one to incorporate existing prior knowledge. In mathematical psychology, parameters typically correspond to psychological variables about which theories exist; the parameter prior can be used to encode these existing psychological theories (e.g., Vanpaemel 2010; Lee and Vanpaemel 2018). Second, proponents of LOO who criticize Bayes factors for being prior dependent do not object to generating predictions based on posterior distributions, as this is an integral part of the LOO procedure. However, the prior that one entertains at a certain time may be the posterior based on past observations. Third, as is good practice in parameter inference, concerns about prior sensitivity of the Bayes factor may be alleviated by conducting sensitivity analyses across a range of plausible prior distributions. In many cases, the sensitivity analysis may show that the qualitative conclusions are robust to the exact prior choice. However, when the results change drastically, this is also valuable information since it highlights that researchers with different, reasonable prior beliefs may draw quite different conclusions.

In sum, we argue that LOO uses the future to predict the past: all data have a temporal structure, even though the analyst may not have access to it or may choose to ignore it. LOO is therefore inconsistent with Dawid’s prequential approach. In contrast, Bayes factors can be naturally conceptualized as assessing the models’ sequential, probabilistic one-step-ahead predictions, and are thus consistent with the prequential approach.

## Rejoinder to Navarro

The commentary by Navarro (this issue) discusses how the scientific goal of explanation aligns with traditional statistical concerns and suggests that the model selection literature may focus too much on the statistical issues of model choice and too little on the scientific questions of interest.^8^ In line with our epistemic view on mathematical psychology, we agree that the starting point should always be meaningful theories that are made precise by implementing them as quantitative models. The models’ plausibilities may then be evaluated based on observed data. In case the data pass what Berkson termed the *interocular traumatic test*—the data are so compelling that the conclusion “hits you straight between the eyes”—no statistical analysis may be required. However, as Edwards et al. (1963, p. 217) remark: “[...] the enthusiast’s interocular trauma may be the skeptic’s random error. A little arithmetic to verify the extent of the trauma can yield great peace of mind for little cost.” Furthermore, often the data may not yield a clear result at first sight; consequently, we believe it is useful to more formally quantify the evidence for the models, just as it is useful to make verbal theories precise by implementing them as quantitative models. Of course, researchers should be aware of the assumptions not only of their models but also of their model evaluation metrics. We agree with Lewandowsky and Farrell (2010, p.10): “Model comparison rests on both quantitative evaluation and intellectual and scholarly judgment.”

Navarro writes, “I am of the view that the behaviour of a selection procedure applied to toy problems is a poor proxy for the inferential problems facing scientists.” First, although the examples we used are simple, we do not regard them as “toy problems.” Our first example dealt with quantifying evidence for a general law of the form “all *X*’s have property *Y* ”; this is perhaps the world’s oldest inference problem and has been discussed by a plethora of philosophers, mathematicians, and statisticians (e.g., Laplace 1829/1902; Polya 1954a, b; Wrinch and Jeffreys 1919). Even Aristotle was already concerned with making inference about a general law (Whewell 1840, p. 294):^9^

“We find that several animals which are deficient in bile are longlived, as man, the horse, and the mule; hence we infer that *all* animals which are deficient in bile are longlived.” (Analytica Priora, ii, 23)

Second, although we agree with Navarro that scientists should also consider more complex problems, we still believe that considering simple problems is invaluable for investigating how model evaluation metrics behave. Suppose one considers a simple example and finds that a model evaluation metric of interest exhibits highly undesirable properties. One could proceed to more complex problems in the hope that these undesirable properties will not be manifest; however, to us, it seems questionable whether this hope is warranted and it may be considerably harder to verify this in the more complex case.

Navarro uses an example to showcase how Bayes factors can “misbehave.” A general law model $\mathcal {M}_{1}$ that asserts that a Bernoulli probability *𝜃* equals 1 is compared to an “unknown quantity” model $\mathcal {M}_{2}$ that assigns *𝜃* a uniform prior. For any data set of size *n* that consists of only successes with the exception of a single failure, the Bayes factor will decisively rule out the general law model $\mathcal {M}_{1}$ in favor of $\mathcal {M}_{2}$.^10^Navarro concludes that the Bayes factor misbehaves since “In real life none of us would choose $\mathcal {M}_{2}$ over $\mathcal {M}_{1}$ in this situation, because from our point of view the general law model is actually “closer” to the truth than the uninformed model.” Navarro furthermore states: “While there are many people who assert that “a single failure is enough to falsify a theory,” I confess I have not yet encountered anyone willing to truly follow this principle in real life.” Indeed, we believe that a single failure is enough to falsify a general law and so did, for instance, Wrinch and Jeffreys (1919, p. 729):

“[...] if for instance we consider that either Einstein’s or Silberstein’s form of the principle of general relativity is true, a single fact contradictory to one would amount to a proof of the other in every case.”

Other examples are provided by Polya (1954a) who discussed how mathematical conjectures are “irrevocably exploded” by a single failure. For instance, the famous Goldbach conjecture holds that every even integer greater than two can be expressed as the sum of two prime numbers. The conjecture has been confirmed for all integers up to 4 × 10^18^.^11^ Yet, the occurrence of a single failure would refute the Goldbach conjecture decisively. Polya (1954a, p. 6) notes how the search for a suitable decomposition of 60 has ended in success (60 = 7 + 53) and explains:

“The conjecture has been verified in one more case. The contrary outcome would have settled the fate of Goldbach’s conjecture once and for all. If, trying all primes under a given even number, such as 60, you never arrive at a decomposition into a sum of two primes, you thereby *explode the conjecture irrevocably* [italics ours].”

Finally, suppose the general law of interest states that “all swans are white.” In case one traveled to Australia and observed a single black swan, to us, the only reasonable conclusion to draw would be that the general law does not hold. We speculate that researchers who believe that in this situation $\mathcal {M}_{1}$ should be favored do not truly entertain a general law model, but an alternative model $\mathcal {M}_{1}^{\ast }$ that states “*almost* all *X*’s have property *Y*.” Under $\mathcal {M}_{1}^{\ast }$, *𝜃* is assigned a prior that is concentrated near 1 but does not completely rule out values very close to 1 (e.g., *𝜃* ∼Beta(*a*,1), with *a* large). This showcases that what has been termed a “misbehavior” of the Bayes factor may be due to the implicit invocation of a third model $\mathcal {M}_{1}^{\ast }$ as a replacement of the general law model $\mathcal {M}_{1}$.

## Rejoinder to Shiffrin & Chandramouli

Shiffrin and Chandramouli (this issue, henceforth SC) argue in favor of comparing non-overlapping model classes using Bayesian inference. Furthermore, SC advocate focusing on interval-null hypotheses instead of point-null hypotheses. Finally, SC demonstrate that comparing non-overlapping hypotheses (where the null is an interval) eliminates the model selection inconsistency of LOO. We believe it is interesting to see that LOO can be made consistent when the models are defined so that the parameter spaces do not overlap, although—as SC state themselves—the result is not completely unexpected.

SC remark that when testing a point-null versus a hypothesis that assigns a continuous prior distribution to the parameter of interest, the “standard” approach of calculating Bayes factors is identical to SCs proposal to consider non-overlapping models (since a single point has measure zero). Therefore, SCs approach only differs in case one does not consider point-null hypotheses. We believe that it may be of interest to consider interval-hypotheses in certain scenarios; in these cases, we agree that defining the models such that the parameter spaces do not overlap can be beneficial (see also Morey and Rouder 2011). However, we also believe that there are situations where it is useful to test point-null hypotheses.^12^

First, we believe that there are situations in which the point-null is exactly true. SC mention an example of testing ESP with coin flipping and argue that the “chance” point-null hypothesis is never exactly true since coins are never perfect and, consequently, will not produce “heads” with probability exactly .5. However, consider the following alternative experiment for testing ESP: Participants are presented with pictures either on the right or left side of the screen and are asked to indicate on which side the next picture will appear. Suppose that exactly half of the pictures are presented on the right, the other half on the left (and the order is randomly permuted). In this scenario, given that we do not believe in ESP, we believe that the point-null—which states that the probability of a correct response is .5—is exactly true.

Second, we believe that testing point-null hypotheses is crucial in all stages of cognitive model development, validation, and application. When developing and validating a model, it is important to show that certain experimental manipulations selectively influence only a subset of the model parameters, whereas the remaining parameters are unaffected. In applications, cognitive models may be used, for instance, to investigate which subprocesses differ or do not differ in clinical subpopulations (cognitive psychometrics, e.g., Riefer et al. 2002). In these applications, researchers are interested in quantifying evidence for a difference (“there is evidence that cognitive process *X* is affected”), but, crucially, also for an invariance or, equivalently, point-null hypothesis (“there is evidence that cognitive process *Y* is *not* affected”).^13^

Third, even in case one does not believe that the point-null hypothesis can be true exactly, it appears that it is still useful to be able to reject at least this “unreasonable” hypothesis. For instance, if one wants to convince a skeptic that a new research finding works, it seems difficult to do so if one cannot even reject a point-null hypothesis which some people argue is never true exactly.

To use SCs proposal in practice, it appears crucial to be able to detect shared model instances (i.e., parameter settings that predict the same outcome distribution). This may not always be straightforward, especially when the two models are defined on different parameter spaces. Consider the comparison between $\mathcal {M}_{1}$ with parameter *𝜃* ∈Θ and $\mathcal {M}_{2}$ with parameter *ξ* ∈Ξ. Suppose one is told that *𝜃* corresponds to a Bernoulli success probability and *ξ* = log (*𝜃*/(1 − *𝜃*)) denotes the log odds with the restriction that *ξ* > 0. In this case, it is straightforward to see that the models share instances (i.e., the restriction *ξ* > 0 corresponds to *𝜃* > .5). Consequently, it appears to us that SC would recommend to eliminate the shared instances and would consider the comparison between $\mathcal {M}_{1}^{\ast }: \theta \le .5$ and $\mathcal {M}_{2}: \xi > 0$. However, in case the models under consideration are more complex cognitive models that feature many parameters, it may not be trivial to detect whether the models share instances.

SC write that their commentary is motivated by “the desire to have statistics serve science, not science serve statistics.” However, to us, it seems that their approach imposes certain constraints on how researchers can act which appears to go against the dictum advanced by SC. Suppose there are two researchers, A and B, who have different hypotheses, $\mathcal {H}_{A}$ and $\mathcal {H}_{B}$, about a phenomenon of interest. These hypotheses happen to overlap. In line with the fact that “statistics should serve science,” we believe that these two researchers should be allowed to compare their hypotheses in their original versions without first altering the hypotheses to the non-overlapping $\mathcal {H}_{A}^{\ast }$ and $\mathcal {H}_{B}^{\ast }$ to fit SCs Procrustean bed of model comparison with non-overlapping model classes. Moreover, it appears that researcher A and B would need to change their hypotheses again in case a third hypothesis $\mathcal {H}_{C}$ is introduced that partially overlaps with the first two hypotheses.

## Concluding Remarks

In this rejoinder to Vehtari et al. (this issue), Navarro (this issue), and Shiffrin and Chandramouli (this issue), we have pointed out further limitations of Bayesian leave-one-out cross-validation. In particular, (1) LOO-based methods such as Bayesian stacking do not align satisfactorily with the epistemic goal of mathematical psychology; (2) LOO-based methods depend on an arbitrary distinction between parameter estimation and model comparison; and (3) LOO-based methods depend on an arbitrary distinction between data that arrive sequentially or “simultaneously.” In line with Lewandowsky and Farrell (2010), we believe that careful model comparison requires both quantitative evaluation and intellectual and scholarly judgment. We personally prefer quantitative evaluation of models based on consistently using Bayes’ rule for both parameters and models (e.g., via the Bayes factor). This approach has the advantage that, in line with the epistemic purpose of mathematical psychology, it enables the quantification of evidence for a set of competing theories that are implemented as quantitative models. Researchers may criticize the specification of an ingredient of Bayes’ rule such as the prior distribution for a particular application. However, once the ingredients have been specified, there is only one optimal way of updating one’s knowledge in light of observed data: the one that is dictated by Bayes’ rule. Alternative methods may be useful in specific circumstances and for specific purposes but—as we illustrated with the case of LOO—they will break down in other settings yielding results that can be surprising, misleading, and incoherent.

## Appendix A: Jevons (1874) on Bayesian Model Averaging

Jevons’ 1874 masterpiece *The Principles of Science* contains the section “Simple Illustration of the Inverse Problem” that showcases how BMA (for prediction) and posterior prediction are identical operations. For historical interest, and out of respect for the clarity of Jevons’ writing, we present the section in full: “Suppose it to be known that a ballot-box contains only four black or white balls, the ratio of black and white balls being unknown. Four drawings having been made with replacement, and a white ball having appeared on each occasion but one, it is required to determine the probability that a white ball will appear next time. Now the hypotheses which can be made as to the contents of the urn are very limited in number, and are at most the following five:—

$$\begin{array}{@{}rcl@{}} \begin{array}{llllll} 4& \text{white}& \text{and}& 0& \text{black}& \text{balls}\\ 3& \quad\text{,,}& \text{,,}& 1 & \text{,,}& \text{,,}\\ 2& \quad\text{,,}& \text{,,}& 2& \text{,,}& \text{,,}\\ 1& \quad\text{,,}& \text{,,}& 3& \text{,,}& \text{,,}\\ 0& \quad\text{,,}& \text{,,}& 4& \text{,,}& \text{,,} \end{array} \end{array} $$

The actual occurrence of black and white balls in the drawings renders the first and last hypotheses out of the question, so that we have only three left to consider. If the box contains three white and one black, the probability of drawing a white each time is $\frac {3}{4}$, and a black $\frac {1}{4}$; so that the compound event observed, namely, three white and one black, has the probability $\frac {3}{4} \times \frac {3}{4} \times \frac {3}{4} \times \frac {1}{4}$, by the rule already given (p. 233).^14^ But as it is indifferent to us in what order the balls are drawn, and the black ball might come first, second, third, or fourth, we must multiply by four, to obtain the probability of three white and one black in any order, thus getting $\frac {27}{64}$. Taking the next hypothesis of two white and two black balls in the urn, we obtain for the same probability the quantity $\frac {1}{2} \times \frac {1}{2} \times \frac {1}{2} \times \frac {1}{2} \times 4$, or $\frac {16}{64}$, and from the third hypothesis of one white and three black, we deduce likewise $\frac {1}{4} \times \frac {1}{4} \times \frac {1}{4} \times \frac {3}{4} \times 4$, or $\frac {3}{64}$. According, then, as we adopt the first, second, or third hypothesis, the probability that the result actually noticed would follow is $\frac {27}{64}$, $\frac {16}{64}$, and $\frac {3}{64}$. Now, it is certain that one or other of these hypotheses must be the true one, and their absolute probabilities are proportional to the probabilities that the observed events would follow from them (see p. 279).^15^ All we have to do, then, in order to obtain the absolute probability of each hypothesis, is to alter these fractions in a uniform ratio, so that their sum shall be unity, the expression of certainty. Now since 27 + 16 + 3 = 46, this will be effected by dividing each fraction by 46 and multiplying by 64. Thus, the probability of the first, second, and third hypotheses are respectively—

$$\frac{27}{46}, \quad \frac{16}{46}, \quad \frac{3}{46}. $$

The inductive part of the problem is now completed, since we have found that the urn most likely contains three white and one black ball, and have assigned the exact probability of each possible supposition. But we are now in a position to resume deductive reasoning, and infer the probability that the next drawing will yield, say a white ball. For if the box contains three white and one black ball, the probability of drawing a white one is certainly $\frac {3}{4}$; and as the probability of the box being so constituted is $\frac {27}{46}$, the compound probability that the box will be so filled and will give a white ball at the next trial, is

$$\frac{27}{46} \times \frac{3}{4} \text{ or} \frac{81}{184}. $$

Again, the probability is $\frac {16}{46}$ that the box contains two white and two black, and under those conditions the probability is $\frac {1}{2}$ that a white ball will appear; hence, the probability that a white ball will appear in consequence of that condition, is

$$\frac{16}{46} \times \frac{1}{2} \text{ or} \frac{32}{184}. $$

From the third supposition, we get in like manner the probability

$$\frac{3}{46} \times \frac{1}{4} \text{ or} \frac{3}{184}. $$

Now since one and not more than one hypothesis can be true, we may add together these separate probabilities, and we find that

$$\frac{81}{184} + \frac{32}{184} + \frac{3}{184} \text{ or} \frac{116}{184} $$

is the complete probability that a white ball will be next drawn under the conditions and data supposed.” (Jevons 1874/1913, pp. 292–294)

In the next section, *General Solution of the Inverse Problem*, Jevons points out that in order for the procedure to be applied to natural phenomena, an infinite number of hypotheses need to be considered: “When we take the step of supposing the balls within the urn to be infinite in number, the possible proportions of white and black balls also become infinite, and the probability of any one proportion actually existing is infinitely small. Hence the final result that the next ball drawn will be white is really the sum of an infinite number of infinitely small quantities. It might seem, indeed, utterly impossible to calculate out a problem having an infinite number of hypotheses, but the wonderful resources of the integral calculus enable this to be done with far greater facility than if we supposed any large finite number of balls, and then actually computed the results. I will not attempt to describe the processes by which Laplace finally accomplished the complete solution of the problem. They are to be found described in several English works, especially De Morgan’s ‘Treatise on Probabilities,’ in the ‘Encyclopædia Metropolitana,’ and Mr. Todhunter’s ‘History of the Theory of Probability.’ The abbreviating power of mathematical analysis was never more strikingly shown. *But I may add that though the integral calculus is employed as a means of summing infinitely numerous results, we in no way abandon the principles of combinations already treated*.[italics ours]” (Jevons 1874/1913, p. 296).

## Appendix B: Coherence of BMA and Bayesian Parameter Inference

Here, we show why BMA yields the same results as Bayesian parameter inference when the set of models is obtained by partitioning a continuous parameter space into non-overlapping intervals, with prior model probabilities set equal to the prior mass in the respective intervals. Given observed data *y*, a parameter of interest *𝜃*,^16^ a corresponding prior distribution *p*(*𝜃*), and likelihood *p*(*y*∣*𝜃*), the posterior distribution for *𝜃* is given by

4 $$ p(\theta \mid y) = \frac{p(y \mid \theta) p(\theta)}{{\int}_{\Theta} p(y \mid \theta) p(\theta) \text{d}\theta},  $$

where Θ denotes the parameter space. The posterior predictive distribution for new data *y*_new_ is given by

5 $$ p(y_{\text{new}} \mid y) = {\int}_{\Theta} p(y_{\text{new}} \mid \theta, y) p(\theta \mid y) \text{d} \theta,  $$

where it is often the case that *p*(*y*_new_∣*𝜃*,*y*) = *p*(*y*_new_∣*𝜃*).

BMA is based on combining the results of different models based on the models’ plausibilities in light of the observed data. We consider the models $\mathcal {M}_{1}, \mathcal {M}_{2}, \ldots , \mathcal {M}_{M}$ that are obtained by partitioning the parameter space Θ into *M* non-overlapping intervals. We denote these non-overlapping intervals by *A*_1_, *A*_2_,…,*A*_*M*_. For instance, when *𝜃* corresponds to a success probability, we could partition Θ = [0, 1] into two intervals *A*_1_ = [0,.5) and *A*_2_ = [.5, 1]. The prior distribution for *𝜃* under each model $\mathcal {M}_{k}$, *k* = 1, 2,…,*M* is obtained by considering the part of *p*(*𝜃*) that corresponds to the interval *A*_*k*_ and then renormalizing the prior density by the prior mass in that subinterval:

6 $$ p(\theta \mid \mathcal{M}_{k}) = \frac{p(\theta)}{C_{k}} \mathbb{I}\left( \theta \in A_{k}\right), $$

where $C_{k} = {\int }_{A_{k}} p(\theta ) \text {d} \theta $ and $\mathbb {I}$ denotes the indicator function. Note that the *M* models differ only in the prior distribution for *𝜃* but not in the likelihood, consequently $p(y \mid \theta , \mathcal {M}_{k}) = p(y \mid \theta )$. Each model’s prior probability $p(\mathcal {M}_{k})$ is set equal to the prior mass that *p*(*𝜃*) assigns to the interval *A*_*k*_:

7 $$ p(\mathcal{M}_{k}) = {\int}_{A_{k}} p(\theta) \text{d} \theta = C_{k}. $$

Given this set-up, the posterior probability for model $\mathcal {M}_{k}$ corresponds to the posterior mass that the “regular” parameter posterior for *𝜃* assigns to the interval *A*_*k*_:

8 $$\begin{array}{@{}rcl@{}} p(\mathcal{M}_{k} \mid y) &=& \frac{p(y \mid \mathcal{M}_{k}) C_{k}}{{\sum}_{j = 1}^{M} p(y \mid \mathcal{M}_{j}) C_{j}} \\ &=& \frac{{\int}_{A_{k}} p(y \mid \theta) \tfrac{p(\theta)}{C_{k}} \text{d}\theta C_{k}}{{\sum}_{j = 1}^{M} {\int}_{A_{j}} p(y \mid \theta) \tfrac{p(\theta)}{C_{j}} \text{d}\theta C_{j}} \\ &=& \frac{{\int}_{A_{k}} p(y \mid \theta) p(\theta) \text{d}\theta}{{\int}_{\Theta} p(y \mid \theta) p(\theta) \text{d}\theta} \\ &=& {\int}_{A_{k}} p(\theta \mid y) \text{d} \theta, \end{array} $$

where we used—in reverse order—the fact that for *b*_2_ ∈ (*b*_1_, *b*_3_), ${\int }_{b_{1}}^{b_{3}} f(x) \text {d}x = {\int }_{b_{1}}^{b_{2}} f(x) \text {d}x + {\int }_{b_{2}}^{b_{3}} f(x) \text {d}x$.

The model-averaged posterior distribution for *𝜃* is obtained as follows:

9 $$\begin{array}{@{}rcl@{}} p_{\text{BMA}}(\theta \mid y) &=& \sum\limits_{k = 1}^{M} p(\theta \mid y, \mathcal{M}_{k}) p(\mathcal{M}_{k} \mid y) \\ &=& \sum\limits_{k = 1}^{M} \underbrace{\tfrac{p(y \mid \theta) \tfrac{p(\theta)}{C_{k}} \mathbb{I}\left( \theta \in A_{k}\right)}{p(y \mid \mathcal{M}_{k})}}_{p(\theta \mid y, \mathcal{M}_{k})} \underbrace{\tfrac{p(y \mid \mathcal{M}_{k}) C_{k}}{{\sum}_{j = 1}^{M} p(y \mid \mathcal{M}_{j}) C_{j}}}_{p(\mathcal{M}_{k} \mid y)} \\ &=& \frac{p(y \mid \theta) p(\theta)}{{\sum}_{j = 1}^{M} p(y \mid \mathcal{M}_{j}) C_{j}} \sum\limits_{k = 1}^{M} \mathbb{I}\left( \theta \in A_{k}\right) \\ &=& \frac{p(y \mid \theta) p(\theta)}{{\sum}_{j = 1}^{M} {\int}_{A_{j}} p(y \mid \theta) \tfrac{p(\theta)}{C_{j}} \text{d} \theta C_{j}}\\ &=& \frac{p(y \mid \theta) p(\theta)}{{\int}_{\Theta} p(y \mid \theta) p(\theta) \text{d} \theta}, \end{array} $$

where we used the fact that any given value for *𝜃* will only fall in one of *A*_*k*_, *k* = 1, 2,…,*M*, hence, ${\sum }_{k = 1}^{M} \mathbb {I}\left (\theta \in A_{k}\right ) = 1$. This shows that the model-averaged posterior *p*_BMA_(*𝜃*∣*y*) is identical to the “regular” parameter posterior (i.e., Eq. 4).

To obtain the model-averaged predictive distribution for new data *y*_new_, we first note that the predictive distribution for model $\mathcal {M}_{k}$ is given by

10 $$\begin{array}{@{}rcl@{}} p(y_{\text{new}} \mid y, \mathcal{M}_{k}) &=& \int p(y_{\text{new}} \mid \theta, y) p(\theta \mid y, \mathcal{M}_{k}) \text{d} \theta \\ &=& \int p(y_{\text{new}} \mid \theta, y)\! \underbrace{\tfrac{p(y \mid \theta) \tfrac{p(\theta)}{C_{k}} \mathbb{I}\left( \theta \in A_{k}\right)}{p(y \mid \mathcal{M}_{k})}}_{p(\theta \mid y, \mathcal{M}_{k})} \text{d} \theta \\ &=& \frac{{\int}_{A_{k}} \!p(y_{\text{new}} \!\mid\! \theta, y) p(y \!\mid\! \theta) p(\theta) \text{d} \theta}{C_{k} p(y \!\mid\! \mathcal{M}_{k})}. \end{array} $$

The model-averaged predictive distribution is

11 $$\begin{array}{@{}rcl@{}} p_{\text{BMA}}(y_{\text{new}} \mid y) &=&\! \sum\limits_{k = 1}^{M} p(y_{\text{new}} \mid y, \mathcal{M}_{k}) p(\mathcal{M}_{k} \mid y)\\ &=&\! \sum\limits_{k = 1}^{M} \underbrace{\tfrac{{\int}_{A_{k}} p(y_{\text{new}} \mid \theta, y) p(y \mid \theta) p(\theta) \text{d} \theta}{C_{k} p(y \mid \mathcal{M}_{k})}}_{p(y_{\text{new}} \mid y, \mathcal{M}_{k})} \underbrace{\tfrac{p(y \mid \mathcal{M}_{k}) C_{k}}{{\sum}_{j = 1}^{M} p(y \mid \mathcal{M}_{j}) C_{j}}}_{p(\mathcal{M}_{k} \mid y)} \\ &=&\!\tfrac{{\sum}_{k = 1}^{M} {\int}_{A_{k}} p(y_{\text{new}} \mid \theta, y) p(y \mid \theta) p(\theta) \text{d} \theta}{{\sum}_{j = 1}^{M} {\int}_{A_{j}} p(y \mid \theta) \tfrac{p(\theta)}{C_{j}} \text{d} \theta C_{j}} \\ &=&\!\frac{{\int}_{\Theta} p(y_{\text{new}} \mid \theta, y) p(y \mid \theta) p(\theta) \text{d} \theta}{{\int}_{\Theta}p(y \mid \theta) p(\theta) \text{d} \theta}\\ &=&\!{\int}_{\Theta} p(y_{\text{new}} \mid \theta, y) p(\theta \mid y) \text{d} \theta. \end{array} $$

This shows that the model-averaged predictive distribution *p*_BMA_(*y*_new_∣*y*) is identical to the “regular” predictive distribution (i.e., Eq. 5).

## Footnotes

1 Throughout this article, we use the terms *model comparison* and *model selection* interchangeably, although it may be argued that there is a subtle difference.

2 Note that the value of the Bayes factor is independent of the prior model probabilities since it quantifies the *change* from prior to posterior model odds. However, although it is independent of the value of the prior model odds, it assumes that, in principle, these could be specified.

3 Relatedly, Wasserman (2000, p. 103) argued: “Second, even when all models are wrong, it is useful to consider the relative merits of two models. Newtonian physics and general relativity are both wrong. Yet, it makes sense to compare the relative evidence in favor of one or the other. Our conclusion would be: under the tentative working hypothesis that one of these two theories is correct, we find that the evidence strongly favors general relativity. It is understood that the working hypothesis that one of the models is correct is wrong. But it is a useful, tentative hypothesis and, proceeding under that hypothesis, it makes sense to evaluate the relative posterior probabilities of those hypotheses.”

4 See also https://bayesianspectacles.org/bayes-factors-for-those-who-hate-bayes-factors/

5 Appendix A contains a fragment from Jevons (1874/1913) that features another example.

6 Note that, in contrast to $\mathcal {M}_{1}, \mathcal {M}_{2}, \ldots , \mathcal {M}_{10}$, the model $\mathcal {M}_{\text {Estimation}}$ does not fix *k* to a single value but allows *k* to vary freely.

7 The stacking weights were obtained using the loo package (Vehtari et al. 2018).

8 One key aspect that is being discussed is the $\mathcal {M}$-open versus $\mathcal {M}$-closed distinction that we have already addressed in a previous section.

9 The authors would like to state that they disagree with the conclusion in this particular example.

10 Note that *n* may be infinity.

11
http://sweet.ua.pt/tos/goldbach.html

12 We have detailed our arguments for why we believe it can be useful to test point-null hypotheses in the following blog posts: https://tinyurl.com/y8org8bt and https://tinyurl.com/ya7cl3cq.

13 Proponents of interval-null hypotheses might argue that the same can be achieved using interval-null hypotheses. However, one would then need to adjust the statement to read “there is evidence that cognitive process *Y* is *almost* not affected.”
